# Identification of the carotenoid cleavage dioxygenase genes and functional analysis reveal *DoCCD1* is potentially involved in beta-ionone formation in *Dendrobium officinale*

**DOI:** 10.3389/fpls.2022.967819

**Published:** 2022-08-04

**Authors:** Yue Wang, Jianchu Xu, Aizhong Liu

**Affiliations:** ^1^Key Laboratory of Economic Plants and Biotechnology, Yunnan Key Laboratory for Wild Plant Resources, Kunming Institute of Botany, Chinese Academy of Sciences, Kunming, China; ^2^Bio-Innovation Center of DR PLANT, Kunming Institute of Botany, Chinese Academy of Sciences, Kunming, China; ^3^Key Laboratory for Forest Resources Conservation and Utilization in the Southwest Mountains of China, Ministry of Education, Southwest Forestry University, Kunming, China

**Keywords:** *DoCCD1*, beta-ionone, carotenoid cleavage dioxygenase, *Dendrobium officinale*, secondary metabolism, apocarotenoids

## Abstract

The carotenoids are the most widely distributed secondary metabolites in plants and can be degraded by carotenoid cleavage dioxygenase (CCD) to form apocarotenoids including an important C13 compound beta-ionone. Volatile beta-ionone can confer the violet and woody fragrance to plant essential oils, flowers, fruits, and vegetables, which therefore has been used in various industries. *Dendrobium officinale* is a traditional medicinal plant. However, there was limited information on the key enzymes involved in the biosynthesis of beta-ionone in *D. officinale*. In the present study, beta-ionone was detected in stems and leaves of *D. officinale* and genome-wide identification and expression profiles of *CCD* genes were subsequently carried out. There were nine DoCCD members in *D. officinale*. According to the phylogenetic relationship, DoCCD proteins were classified into six subfamilies including CCD1, CCD4, CCD7, CCD8, nine-cis-epoxycarotenoid dioxygenase (NCED) and zaxinone synthase (ZAS). *DoCCD* genes showed distinctive expression profiles and *DoCCD1* gene was abundantly expressed in eight tissues. Induced expression of *DoCCD1* gene resulted in discoloration of *Escerichia coli* strains that can accumulate carotenoids. Analysis of Gas Chromatography/Mass Spectrometer showed that DoCCD1 enzyme can cleave lycopene to produce 6-methyl-5-hepten-2-one and pseudoionone and also catalyze beta-carotene to form beta-ionone. Expression of *DoCCD1* gene in *Nicotiana benthamiana* leaf resulted in production of abundant beta-ionone. Overall, the present study first provides valuable information on the CCD gene family in *D. officinale*, function of *DoCCD1* gene as well as production of beta-ionone through genetic modification.

## Introduction

Carotenoids are C40 isoprenoids that are the most widely distributed secondary metabolites in plants. Due to abundant double bonds, carotenoids tend to be oxidized through catalytic action by enzymes in plants or induction by ROS, which results in formation of various products that are named as apocarotenoids (Auldridge et al., 2006a; Moreno et al., 2021). Apocarotenoids include important hormones abscisic acid (ABA), strigolactones as well as other small molecules such as ionone, zaxinone, anchorene (Felemban et al., 2019). These apocarotenoids have been shown to play important roles in plant growth, development, response to various stresses and signaling communication (Felemban et al., 2019; Moreno et al., 2021). Among them, volatile compound beta-ionone (C13) has violet and woody notes, which can confer aromas to flowers, fruits, and vegetables (Paparella et al., 2021). Moreover, pharmacological activities of beta-ionone have been confirmed (Aloum et al., 2020; Paparella et al., 2021). With approved security, beta-ionone has been used in the food and fragrance industries (Aloum et al., 2020). Due to its importance and wide application, the biosynthesis of beta-ionone has attracted great attentions.

In plants, carotenoids can be catalyzed by carotenoid cleavage dioxygenases (CCDs) at specific bond sites to produce various apocarotenoids, including aromatic volatile beta-ionone (Simkin et al., 2004b). CCD protein as non-heme iron enzymes is usually characteristics of four conserved histidines and a conserved peptide sequence at the C-terminus (Auldridge et al., 2006a). Based on the substrate difference, the CCD family consisting of multiple members in plants can be classified into CCD and 9-cis-Epoxycarotenoid Dioxygenase (NCED) types (Tan et al., 2003). According to different functions, CCD can be further divided into different subfamilies, such as CCD1, CCD4, CCD2, CCD7, CCD8, and ZAS. In Arabidopsis, there are nine CCD members, including five NCEDs (NCED2, NCED3, NCED5, NCED6, and NCED9) and four CCDs (CCD1, CCD4, CCD7, and CCD8) (Tan et al., 2003). Classification of CCDs in other plants is mainly based on the study in Arabidopsis and some CCD members with new functions were found in other plants such as CCD2 in crocus and *Freesia hybrida* (Ahrazem et al., 2016a; Fang et al., 2020), ZAS in rice (Wang et al., 2019).

Although CCD enzymes can cleave carotenoids, the CCD enzymes from different subfamilies show great differences in substrates and cleavage sites, and therefore result in function diversification (Auldridge et al., 2006a). The first identified CCD was Viviparous 14 in maize (Schwartz et al., 1997). Viviparous 14 and its homologs in Arabidopsis NCEDs can cleave 9-cis-violaxanthin or 9-cis-neoxanthin at 11, 12 (11′, 12′) sites and directly participate in the biosynthesis of ABA (Schwartz et al., 1997; Tan et al., 2003). CCD2 was found in limited plants such as crocus and *Freesia hybrida* and can degrade zeaxanthin at 7, 8, or 7′, 8′ sites for the biosynthesis of safranal and crocin (Frusciante et al., 2014; Ahrazem et al., 2016a; Fang et al., 2020). CCD7 and CCD8 are mainly related to the formation of strigolactones (Alder et al., 2012). ZAS can produce zaxinone with 3-OH-β-apo-10′-carotenal as the substrate, and control the contents of strigolactones in Arabidopsis and rice (Wang et al., 2019; Ablazov et al., 2020).

**FIGURE 1:**
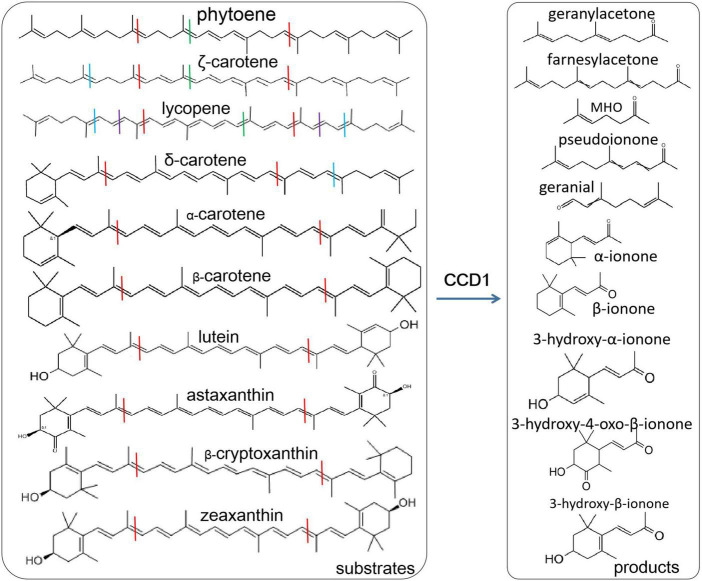
Various carotenoid substrates and products by CCD1 cleavage. Lines with different colors indicate the cleavage sites, blue at 5, 6 and 5′, 6′; red at 9, 10 and 9′, 10′; dark red at 13, 14 and 13′, 14′; purple at 7, 8, and 7′, 8′.

The functions of CCD1 and CCD4 have been deeply investigated and their substrates are mainly carotenoids. However, the cytoplasmic CCD1 has more abundant substrates, including zeaxanthin, lutein, etc. (Auldridge et al., 2006a; Vogel et al., 2008; Ahrazem et al., 2016b; Zheng et al., 2021; Figure 1). The plastid-located CCD4 has higher substrate specificity (Us-Camas et al., 2022). CCD1 and CCD4 cleave carotenoids to increase the content of apocarotenoids and therefore have important influences on plant quality, such as the carotenoid contents in crops (Ko et al., 2018; Thakur et al., 2021), the color and flavors in vegetables and fruits (Campbell et al., 2010; Fukamatsu et al., 2013; Ilg et al., 2014), the flower color and fragrance (Ohmiya et al., 2006; Huang et al., 2009; Chiou et al., 2010; Zhang et al., 2015; Gao et al., 2021) and tea quality (Wang et al., 2020a,b). CCD1 in tomato was responsible for the formation of volatile isoprenes including beta-ionone, pseudoionone, and geranylacetone (Simkin et al., 2004a). CCD1 and CCD4 in *Bixa orellana* were recently found to be involved in the bixin biosynthesis (Us-Camas et al., 2022). Moreover, CCD1 have great effects on the emission of beta-ionone. PhCCD1 can control the emission of beta-ionone in petunia flower (Simkin et al., 2004b) and the similar phenomena were also found in other plants such as *Osmanthus fragrans* (Baldermann et al., 2010), tomato (Simkin et al., 2004a), tea (Wang et al., 2020a), Brassica species (Zhang et al., 2015).

*Dendrobium officinale* is a traditional medicinal plant (Ng et al., 2012). Modern studies have shown that there are abundant bioactive compounds, such as alkaloids, bibenzyl, polysaccharides, and phenolic compounds (Tang et al., 2017; Wang Y. et al., 2022). However, there is limited knowledge on the biosynthesis of beta-ionone. In the present study, beta-ionone was detected in stems and leaves through headspace GC/MS and then genome-wide identification and characterization of the CCD gene family were performed in *D. officinale*. The expression of *DoCCD* genes was analyzed in various tissues and the function of DoCCD1 was investigated in *E. coli* as well as in plants. These results provide the valuable information on the CCD gene family as well as the function of DoCCD1 in the biosynthesis of beta-ionone.

## Materials and methods

### Plant materials

12-month-old plants *in vitro* of *Dendrobium officinale* Kimura et Migo were grown in the tissue culture room of the Kunming Institute of Botany, Chinese Academy of Sciences (Kunming, Yunnan, China). The growth condition was at 25 ± 2°C with a photoperiod of 12 h light/12 h dark. Healthy leaves and stems were harvested, quickly frozen in liquid nitrogen and kept at −80°C for future RNA extraction. After 50 days grown in soils, *Nicotiana benthamiana* plants were used to *Agrobacteria*-mediated injection.

### Identification and phylogenetic analysis of carotenoid cleavage dioxygenase genes in *Dendrobium officinale*

The RPE65 (retinal pigment epithelial membrane protein) domain was used to search the putative CCD proteins in *D. officinale* genome (Zhang et al., 2016) with HMMER (Finn et al., 2015) and E value < 1e^–100^. After removing the repeated sequences, the sequences were remained with the conserved domain confirmed by SMART and NCBI. The isoelectric point and molecular weight of DoCDD deduced proteins were calculated with the ProtParam tool (Wilkins et al., 1999). Potential localization of DoCDD proteins was predicted by online iPSORT Prediction^1^ (Bannai et al., 2002). Moreover, the sequences of nine *Arabidopsis thaliana AtCCD* genes were obtained from TAIR and the sequences of 13 rice (*Oryza sativa*) *OsCCD* genes were obtained from phytozome.^2^ The sequences of CCD proteins from three species were aligned through ClustalW (McWilliam et al., 2013) and a phylogenetic tree was constructed using MEGA 7.0 (Kumar et al., 2016) with the neighbor-joining method and a bootstrap of 1,000 replicates.

### Conserved motifs and gene structures of *DoCCD genes*

Ten conserved motifs embedded in the CDD proteins were searched with MEME^3^ (Bailey et al., 2009) and potential functions of these motifs were further analyzed through the InterPro database^4^ (Apweiler et al., 2001). The gene structure of *CCD* genes was obtained by comparing the genomic sequences with the coding sequences with online GSDS^5^ (Hu et al., 2015).

### Expression analysis of *DoCCD* genes in various tissues

The expression patterns of nine *DoCCD* genes were analyzed based on the previously published transcriptomic data (PRJNA348403, Zhang et al., 2017). Eight tissues included column, flower buds, green root tip, leaf, lip, sepal, stem, and white root. The FPKM method was used to estimate the transcript abundance (Wang and Liu, 2020) and a heatmap was obtained through R package pheatmap (v1.0.10).^6^

### Amplification of *DoCCD1* gene

The total RNA was extracted from leaf sample using the RNAprep Pure Plant Plus Kit (Cat DP441, Tiangen, China). The cDNA was obtained with the one-step cDNA Synthesis Kit (Cat AT311, Transgen, China). The coding fragments were amplified using high fidelity Q5 polymerase (NEB) with the specific primers of *DoCCD1* gene. The sequences of forward and reverse primers were 5′-ATGGAGAAGGAAAATGGAA-3′ and 5′-CTTTCCCTGTTGTTGAAGTTGTTCC-3′, respectively.

### Expression of *DoCCD1* gene in *Escerichia coli* strains

The *DoCCD1* fragment was cloned into the pThio-Dan1 vector through the ClonExpress II One Step Cloning Kit (Vazyme, Nanjing, China), which produced the vector pThio-Dan1-DoCCD1. *DoCCD1* gene was driven under the arabinose-induced promoter. The expression vector pThio-Dan1-DoCCD1 and the empty vector pThio-Dan1 were transformed into different *E. coli* strains BL21-AI, respectively. The BL21-AI strains carry different carotenoid-producing genes and therefore accumulate different types of carotenoids, including lycopene (pACCRT-EIB), beta-carotene (pACCAR16△crt) and zeaxanthin (pACCAR25△crtX) (Misawa et al., 1995). When the OD_600_ value of cultures was up to 0.6, 0.1 mM arabinose was added. The strains were cultured at 16°C for 20 h to induce protein expression and strain cultures were harvested for further analysis.

### Transient expression of *DoCCD1* gene in *Nicotiana benthamiana*

The *DoCCD1* fragment was cloned into plant expression vector pHREAC through the ClonExpress II One Step Cloning Kit (Vazyme, Nanjing, China), therefore producing pHREAC-DoCCD1. *DoCCD1* gene was driven under 35S promoter. The empty vector pHREAC and the expression vector pHREAC-DoCCD1 were transformed into *Agrobacteria* strain GV3101, respectively. *N. benthamiana* leaves were injected, as described by Bruückner and Tissier (2013). Briefly, the *Agrobacteria* were cultured and then resuspended with infiltration buffer with OD_600_ of 0.5. The plants were infiltrated with 1 mL syringe on the abaxial side of leaves and maintained at the growth chamber. After 3 days leaves were harvested, immediately frozen and maintained at −80°C for further analysis.

### Headspace solid-phase microextraction-gas chromatograph/mass spectrometer for volatile analysis

Fresh plant materials after ground in liquid nitrogen or strain cultures were used for headspace volatile analysis. The solid-phase microextraction (SPME) was connected to the headspace bottle and the samples were treated at 40°C for 30 min for volatile collection. The analysis of volatiles was carried out by Agilent7890-5975 Gas Chromatograph/Mass Spectrometer (GC/MS). The separation column was DB-5MS (30 m × 0.25 mm × 0.25 μm) and helium was used as the carrier gas with a flow velocity of 1.1 mL/min. The temperature at the sample inlet was 250°C. The GC oven was first maintained at 40°C for 1 min, and then followed by 2°C/min up to 60°C and finally 10°C/min up to 325°C. The temperature of ion source was kept at 250°C. The MS data was acquired through scanning the range of m/z 33–500. The products were qualitatively analyzed based on the retention index (RI) and mass spectra of standards.

### Analysis of carotenoids by high performance liquid chromatography

Carotenoids produced in 50 mL *E. coli* strain cultures were obtained through extraction with sonication-mediated acetone. The analysis of 5 μL sample was carried out on the Waters e2695 Alliance High Performance Liquid Chromatography (HPLC). The column was WaterNova-PAK-C18 (3.9 m × 150 mm × 4μm) at 30°C. The absorbance was detected at 450 nm. The separation was performed a flow rate of 0.5 mL/min by isopropanol as solvent A and acetonitrile:water (8:2, v/v) as solvent B. The gradient elution was used as following 100% B (0 min); 100% A (40 min). The equilibrium time was 6 min. Peaks were qualitatively identified according to the retention index of standards in the database.

## Results

### Abundant beta-ionone in *Dendrobium officinale*

**FIGURE 2:**
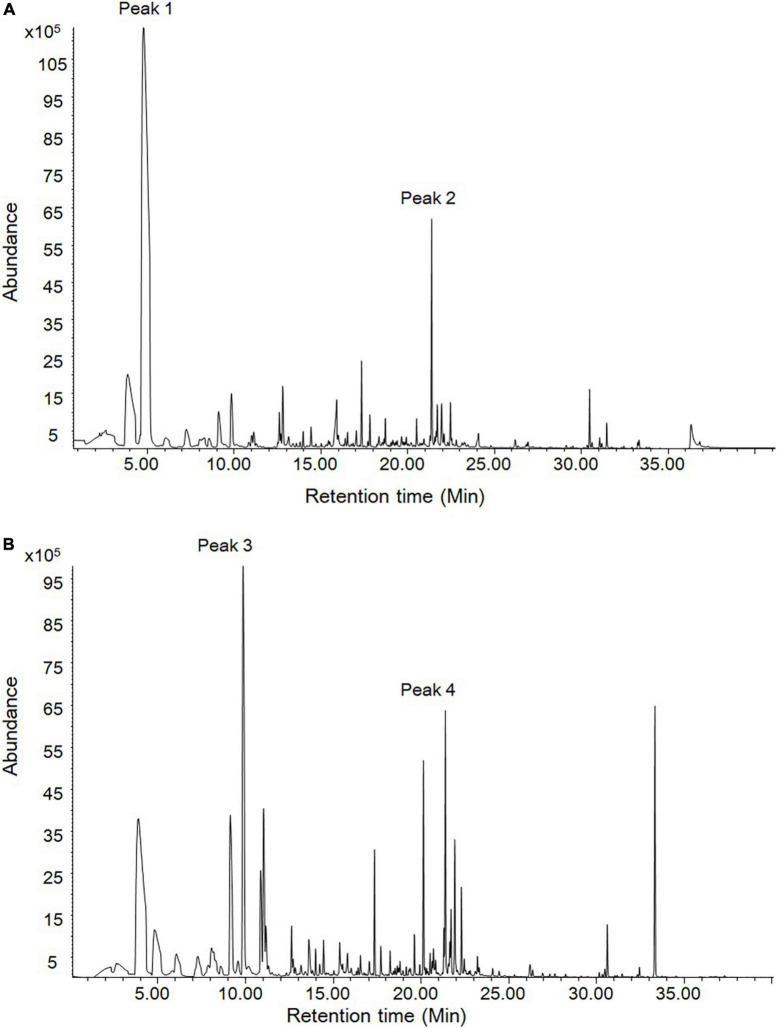
The volatile compounds in leaves and stems of *D. officinale* through headspace SPME-GC/MS analysis. **(A)** The leave volatiles were detected. **(B)** The stem volatiles were detected. According to RI and mass spectra, peak 2 and peak 4 were qualitatively identified as beta-ionone.

The stems and leaves of *D. officinale* are main resource materials for medicines and foods. Therefore, their volatile compounds were detected through headspace SPME-GC/MS. As shown in Figure 2, it was found that there were two and three most significant peaks in leaf and stem samples, respectively. According to the RI and mass spectra, four peaks (1–4) were qualitatively identified (Supplementary Figure 1). The most abundant compound in leaf was 2-hexenal (peak 1) that has fresh green leaf notes while the most abundant compound in stem was 2-octenal (peak 3). Importantly and interestingly, beta-ionone was found to be the commonly dominant compound of volatiles in both the leaf and stem (peak 2 and peak 4) and the levels in two tissues were similar. The beta-ionone with violet and woody notes has been widely used in various industries, which is therefore worth further investigating on its biosynthesis in *D. officinale*.

### Identification and phylogenetic analysis of *DoCCD* members in *Dendrobium officinale*

CCD proteins have a typical domain, namely, RPE65 (retinal pigment epithelial membrane protein) domain. To obtain the potential genes are potentially involved in the production of beta-ionone in *D. officinale*, identification of *CCD* genes was first carried out based on its genome using RPE65 domain as query with *E*-value < 1e^–100^ through HMM3.0. After removal of repeated sequences and confirmation of present RPE65 domain, a total of nine CCD candidates were obtained. According to the homologs in Arabidopsis and rice, these CCD members were named as DoCCD1, DoCCD4, DoCCD7a, DoCCD7b, DoCCD7c, DoCCD8, DoNCED2, DoNCED3, and DoZAS. The detailed information was listed in Supplementary Table 1. The lengths of coding sequences ranged from 750 to 1,902 bp, while the lengths of genomic DNA sequences varied from 750 to 4,1354 bp. The lengths of putative proteins were 249–633 AA, with molecular weights of 27.16–71.81 kD and pI of 5.97–9.39. CCD proteins usually include a chloroplast transit peptide (cTP) domain at the N-terminal, which can facilitate proteins to the plastid (Tan et al., 2003). The prediction of the cTP domain showed that seven DoCCD proteins contain the cTP domain while DoCCD1 and DoCCD4 lack the cTP domain (Supplementary Table 1).

**FIGURE 3:**
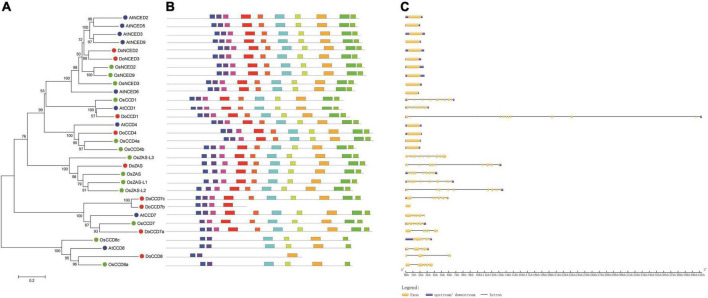
Phylogenetic analysis and characterization of *CCD* genes from three species. **(A)** The phylogenetic tree of 31 CCD proteins. CCD proteins were aligned with ClustalW and an unrooted tree was constructed using MEGA 7.0 with the neighbor-joining method and bootstraps of 1,000 replicates. **(B)** Ten conserved motifs were analyzed in CCD proteins. **(C)** The exon-intron structures of *CCD* genes were analyzed. *At, Do* and *Os* stand for *Arabidopsis thaliana, Oryza sativa* and *Dendrobium officinale*, respectively.

To reveal the relationship between CCD proteins, a phylogenetic tree was constructed including nine DoCCDs, nine AtCCDs, and 13 OsCCDs (Figure 3A). These CCD proteins were clearly classified into six subfamilies, including CCD1, CCD4, CCD7, CCD8, ZAS, and NCED, which was the same as in rice (Wang et al., 2019) and *Gossypium* (Zhang et al., 2021). In *D. officinale*, there were three members in the CCD7 subfamily while there was only one member in Arabidopsis and rice, respectively. DoCCD7a was close to AtCCD7 and OsCCD7 proteins whereas DoCCD7b and DoCCD7c formed a new branch. There were great differences in the number of NCED members among species, such as five in Arabidopsis, three in rice, and two in *D. officinale*. For other four subfamilies, there was only one member in *D. officinale*. The ZAS subfamily was newly identified (Wang et al., 2019). There were one and four members in *D. officinale* and rice, respectively, while no member was found in Arabidopsis. These results showed CCD proteins are conservatively present in plants and may maintain their conserved functions.

### Conserved motifs and gene structure of *DoCCD* genes

To further characterize *DoCCD* genes, the gene structure and conserved motifs were analyzed. It was found that there were 10 conserved motifs, representing part of the RPE65 domain (Figure 3B). Most of the members (25) contained all the 10 motifs, while other members lacked some motifs, such as DoCCD7b and OsCCD7. Members from the CCD8 subfamily contained only five or six motifs. The analysis of gene structures showed that there were notable differences between subfamilies while the members in the same subfamily showed the same or similar gene structures (Figure 3C). The members from the NCED and CCD4 subfamilies were intronless, while other members contained varying introns. All the members from the CCD1 and ZAS subfamilies contained 12 introns, six introns were embedded in CCD8 members while most members of the CCD7 subfamily included six introns, expect *DoCCD7b* having no intron. Notably, although *CCD* genes in the same subfamily had the same number of introns, the introns in *DoCCD* genes were generally longer than those in Arabidopsis and rice. These results suggest that *CCD* genes maintain highly conserved gene structures during evolution.

### Expression patterns of *DoCCD* genes in various tissues

**FIGURE 4:**
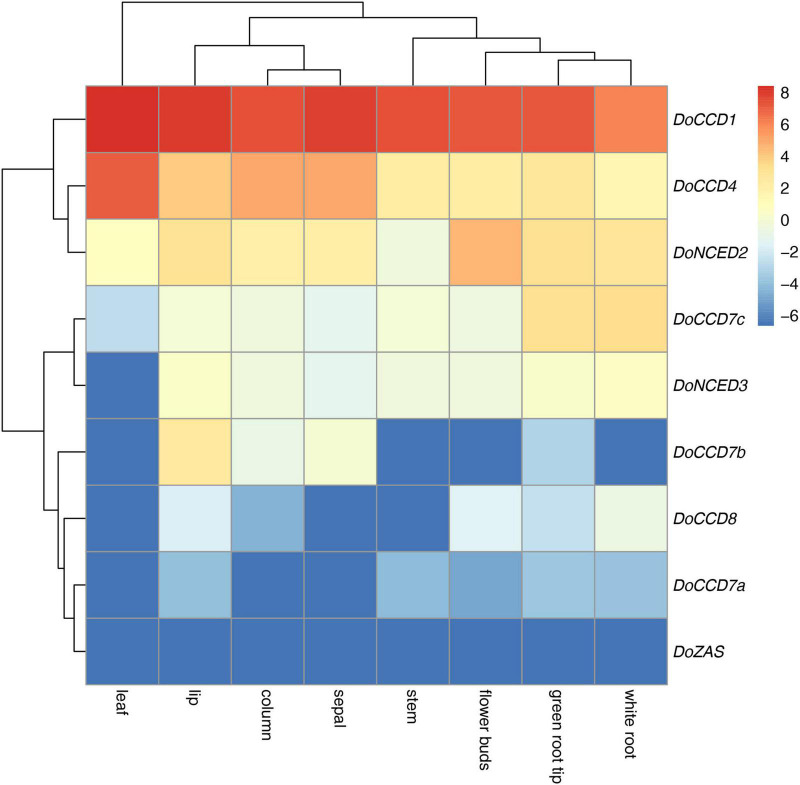
Expression heatmap of nine *DoCCD* genes in eight tissues. The data was from the RNA-Seq (Zhang et al., 2017) including tissues such as column, flower buds, lip, sepal, leaf, stem, white root, and green root tip. R package was used to construct the heatmap and the color scale stands for the normalized values.

The patterns of gene expression can provide important information for their functions. According to the previously transcriptomic data (Zhang et al., 2017), nine *DoCCD* genes were analyzed in eight tissues, including column, flower buds, green root tip, leaf, lip, sepal, stem, and white root. As shown in Figure 4, these genes exhibited distinct expression profiles. Among them, *DoCCD7a, DoCCD8*, and *DoZAS* genes were hardly detected in these tissues. *DoCCD7b* was expressed in column, flower buds and lip with varying levels and the transcripts in lip were the most abundant. *DoCCD7c* gene was almost not expressed in six tissues, but specifically and highly expressed in green root tip and white root. Two *DoNCED* genes showed differential expression patterns. *DoNCED3* gene was expressed with very low or no detectable levels while *DoNCED2* gene was expressed in various tissues with low levels. The highest transcript of *DoNCED2* gene was in flower buds and the second was in root, suggesting their divergent functions. *DoCCD4* gene was extensively expressed in eight tissues, and the transcripts in leaf were the most abundant with an FPKM of 137.10 and followed by sepal and column. *DoCCD1* gene was highly and extensively detected in eight tissues and the highest transcript was found in leaf with an FPKM of 338.06 and the lowest transcript in the white root with an FPKM of 68.37.

### Function analysis of *DoCCD1* enzyme

**FIGURE 5:**
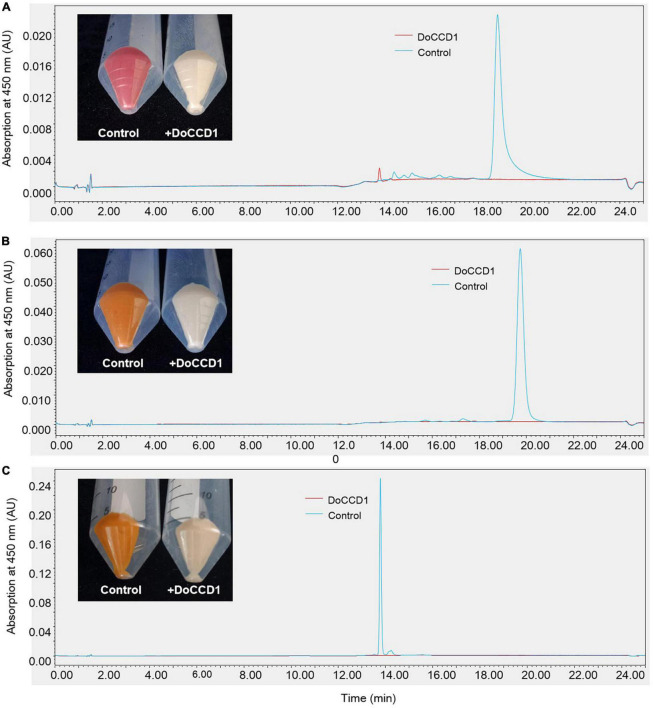
The color changes and HPLC analysis of *E. coli* strains. **(A)** *E. coli* strain can accumulate lycopene (pACCRT-EIB). **(B)** *E. coli* strain can accumulate beta-carotene (pACCAR16△crt). **(C)** *E. coli* strain can accumulate zeaxanthin (pACCAR25△crtX). Control stands for the strain containing empty vector pThio-Dan1 while + DoCCD1 stands for the strain containing plasmid pThio-Dan1-DoCCD1.

Due to low solubility of carotenoids in water, it is usually difficult to carry out experiments *in vitro*. An alternative is to detect the CCD activity in *E. coli* strains that can accumulate the specific type of carotenoids. To detect potential function of the enzyme encoded by *DoCCD1* gene, the CDS sequences were cloned to produce an inducible pThio-Dan1-DoCCD1 vector. The expression of *DoCCD1* gene embedded in this vector can be induced once arabinose is added into the culture solution. The plasmid pThio-Dan1-DoCCD1 and empty vector pThio-Dan1 were transformed to three BL21-AI strains that can accumulate lycopene (pACCRT-EIB), beta-carotene (pACCAR16△crt), and zeaxanthin (pACCAR25△crtX) (Misawa et al., 1995), respectively. After induced expression of *DoCCD1* gene by arabinose, it was found that the strain cultures exhibited remarkable changes in colors, varying from lighter color to almost white (Figure 5), while the controls (empty vector) kept pigmentation. These results suggest that *DoCCD1* gene was functional in *E. coli* and DoCCD1 enzyme may use multiple substrates such as lycopene, beta-carotene, and zeaxanthin, which then can result in lack of carotenoid pigmentation. The carotenoids extracted from these strain cultures were further detected by HPLC. As shown in Figure 5, abundant carotenoids (lycopene, beta-carotene, and zeaxanthin) were found in the control cultures while almost no abundance was detected in the strain cultures with expression of *DoCCD1* gene. These results demonstrate that DoCCD1 can catalyze these three carotenoids to result in color changes.

**FIGURE 6:**
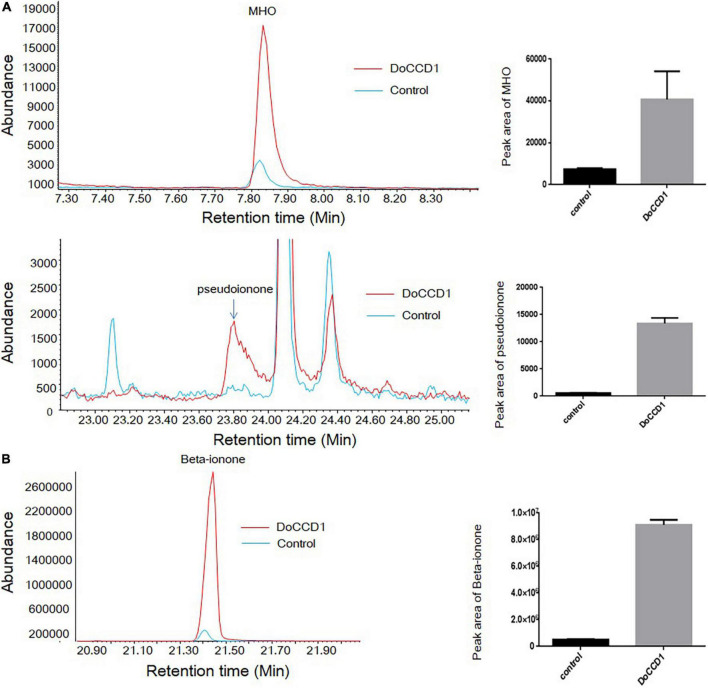
Products in carotenoid-producing *E. coli* strains by headspace SPME-GC/MS. **(A)** *E. coli* strains can accumulate lycopene. Two peaks were identified as MHO (up) and pseudoionone (down), respectively. **(B)** *E. coli* strains can accumulate beta-carotene. The peak was identified as beta-ionone. Red lines stand for the strain containing *DoCCD1* gene. Blue lines stand for the control strain without *DoCCD1* gene.

To determine the possible products in carotenoid accumulation strains with expression of *DoCCD1* gene, the volatiles were detected by headspace SPME-GC/MS (Figure 6). After *DoCCD1* gene was expressed in the strains that can yield lycopene, two new peaks were remarkably observed, but their abundances were low in the control (Figure 6A). According to the RI and mass spectra in the database, these two products were qualitatively identified as pseudoionone and 6-methyl-5-hepten-2-one (MHO), respectively. These results demonstrate that DoCCD1 can cleave the lycopene at different bond sites. Pseudoionone was formed through the cleavage at 9, 10 and 9′, 10′ sites while MHO was formed through the cleavage at 5, 6 and/or 5′, 6′ sites. Notably, the abundance of MHO was much higher than that of pseudoionone, which implies that more lycopene was cleaved at 5, 6 and/or 5′, 6′ sites by DoCCD1. After *DoCCD1* gene was expressed in the beta-carotene accumulation strains, a new peak was obviously observed, but the peak was much low in the control (Figure 6B). The product was qualitatively identified as beta-ionone based on RI and mass spectra. These results demonstrate that DoCCD1 can catalyze beta-carotene at the 9, 10 and 9′, 10′ bond sites to produce beta-ionone. After *DoCCD1* gene was expressed in zeaxanthin accumulation strains, no significant peak was detected compared with the control, although color change was found. These results suggest that DoCCD1 may cleave zeaxanthin but produce undetectable products by GC/MS.

**FIGURE 7:**
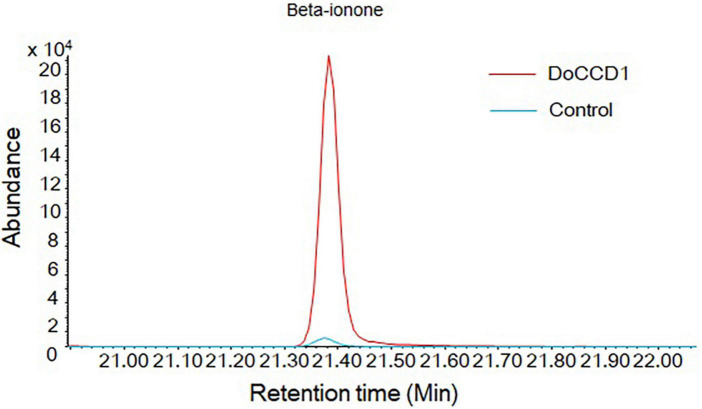
Products in *N. benthamiana* leaves with expression of *DoCCD1* by headspace SPME-GC/MS. The peak was identified as beta-ionone. The red line stands for *N. benthamiana* leaves with expression of *DoCCD1* gene. The blue line stands for control leaves without *DoCCD1* gene.

To determine the function of *DoCCD1* in *planta*, the fragment of *DoCCD1* gene was cloned into plant expression vector pHREAC and under 35S promoter, producing engineered GV3101 strain. The volatiles in *N. benthamiana* leaves were analyzed by headspace SPME-GC/MS (Figure 7). The results showed that a remarkable peak appeared in the *N. benthamiana* leaves with *DoCCD1* expression, while the peak in the control was very low. This peak was qualitatively to be beta-ionone. These results suggest that *DoCCD1* gene was functional in *N. benthamiana* leaf and DoCCD1 enzyme can catalyze the *N. benthamiana* carotenoids to produce beta-ionone.

## Discussion

Carotenoids and their derivatives apocarotemoids play important roles in plant life including growth, development and responses to environmental changes. Among them, beta-ionone, a C13 volatile compound with a relatively low odor threshold is one of the key components for plant essential oils, flower and fruit flavors (Huang et al., 2009; Baldermann et al., 2010; Paparella et al., 2021). The bioactive effects of beta-ionone such as anti-cancer have been demonstrated (Paparella et al., 2021). *D. officinale* has been used as a medicinal, edible, and ornamental plant, and is rich in polysaccharides, phenols, and terpene compounds (Tang et al., 2017; Wang Y. et al., 2022). In our present study, among the volatile components in the stem and leaf, the relatively abundant beta-ionone was found (Figure 2), which was similar to the previous report (Ma et al., 2018). Beta-ionone was also detected in the fresh flowers of *D. loddigesii* (Wang S. et al., 2022). No beta-ionone was detected in some reports (Chen et al., 2016; Hu et al., 2020), which might be due to the different methods applied. The headspace SPME-GC/MS method used in the present work is effective to detect beta-ionone. The presence of beta-ionone in *D. officinale* can provide important basis for use of leaves and stems and their effective values.

The *CCD* genes encode the CCDs that can catalyze carotenoids into formation of apocarotenoids, such as beta-ionone. In our study, a total of nine *CCD* gene members were first identified in *D. officinale*, which can be classified into six subfamilies including CCD1, CCD4, CCD7, CCD8, NCED, and ZAS as in rice (Wang et al., 2019). However, Arabidopsis lacks the ZAS subfamily (Tan et al., 2003). The numbers of *CCD* genes in different species vary greatly, which could be related to the genome ploidy, gene replication and species evolution. There were nine *CCD* gene members in *D. officinale* and Arabidopsis (Tan et al., 2003), respectively, which were less than the CCD members in other species, such as rice (Tan et al., 2003), tobacco (Zhou et al., 2019), cotton (Zhang et al., 2021), Saccharum (Su et al., 2021). In dicotyledonous plants Arabidopsis and cotton, there were more NCED members than those of the CCD type, suggesting the expansion of NCED and their important roles. However, in monocotyledonous plants *D. officinale* and rice, there were only two or three NCED members. These results imply that duplication of *NCED* genes may happen after differentiation of the monocot and eudicot (Vallabhaneni et al., 2010). Notably, there were three members in the CCD7 subfamily in *D. officinale*. Among them, DoCCD7a was closely clustered with AtCCD7 and OsCCD7, suggesting their conserved function. However, DoCCD7b and DoCCD7c formed a new branch, which may imply their new unknown functions. The analysis of gene structures revealed that the *DoCCD* genes from the same subfamily shared the same or similar patterns while the *DoCCD* genes from the different subfamilies exhibited different patterns (Figure 3). The genes from the NCED and CCD4 subfamilies had no intron while other *DoCCD* genes included varying numbers of introns. The similar profiles have been observed in Arabidopsis and rice (Figure 3). The intronless genes usually showed a faster evolution rate, which can facilitate their quick response to the environmental changes (Tan et al., 2003; Zhang et al., 2021).

The patterns of gene expression can provide important information for their roles in plants. *DoCCD* genes showed distinct expression profiles (Figure 4). *DoCCD1* gene was expressed in various tissues, as other *CCD1* genes from other plants, including Arabidopsis (Auldridge et al., 2006b), tomato (Simkin et al., 2004a), petunia (Simkin et al., 2004b), strawberry (García-Limones et al., 2008), cotton (Zhang et al., 2021), which were expressed in leaf, flower, stem, fruit, and root. The extensive expression of *CCD1* genes in plant tissues shows their conserved function. The high expression of *CCD1* gene in flower and fruit can enhance emission of volatile apocarotenoids, which therefore was helpful to attract pollinators and propagators (García-Limones et al., 2008). The expression of *CCD1* genes in vegetative tissues can produce some apocarotenoids with antimicrobial activities, which can prevent plants from infection of fungal pathogens (Simkin et al., 2004b; Auldridge et al., 2006b). *CCD7* and *CCD8* genes were mainly expressed in the roots of rice, Arabidopsis, and petunia (Vallabhaneni et al., 2010) and involved in the biosynthesis of strigolactone (Alder et al., 2012). In *D. officinale*, three *DoCCD7* genes showed differential expression patterns. Among them, *DoCCD7c* gene was specifically expressed in root tissues, indicating its possible roles in the biosynthesis of strigolactone. However, *DoCCD7b* gene was expressed in some flower tissues, implying its putative functions in flower development. The *NCED* genes are mainly related to the biosynthesis of ABA (Schwartz et al., 1997). *DoNCED3* was not expressed while *DoNCED2* gene was expressed with low levels in various tissues, which suggests that the function of DoNCED3 may be lost while DoNCED2 may mainly play roles in the formation of ABA in root and unknown roles in flowers.

Previous studies have shown that CCD1 enzyme can utilize a series of compounds as substrates, including acyclic and cyclic carotenoids (Vogel et al., 2008). Due to low solubility of carotenoids in water, the activity of CCD1 enzymes was mainly performed on *E. coli* strains that can accumulate different carotenoids (Misawa et al., 1995). In our study, DoCCD1 can cleave three substrates (lycopene, beta-carotene, and zeaxanthin) to result in discoloration (Figure 5). When zeaxanthin was used as the substrate, no product was detected by GC/MS or HPLC, which has been observed before (Auldridge et al., 2006b). The possible explanation was the volatile ability of products was too low or the products were catabolized by the strains (Auldridge et al., 2006b). Previous studies have shown that CCD1 can cleave the substrates at the 9, 10 (9′, 10′) bond sites, such as CCD1 from *A. thaliana* (Schwartz et al., 2001), tea plant (Wang et al., 2020a), *Morus notabilis* (Qi et al., 2021), *Petunia hybrida* (Simkin et al., 2004b), *Lycopersicon esculentum* (Simkin et al., 2004a), *Rosa damascena* (Huang et al., 2009), *Osmanthus fragrans* (Baldermann et al., 2010). When beta-carotene was the substrate for CCD1, the cleavage at the 9, 10 (9′, 10′) bond sites can produce beta-ionone (Schwartz et al., 2001), which can influence the fragrance and flavors of flowers, fruits, and essential oils (Schwartz et al., 2001; Simkin et al., 2004a,b; Brandi et al., 2011). When lycopene was the substrate, CCD1 enzymes from different species showed various cleavage sites. DoCCD1 can cleave lycopene at 5, 6 and/or 5′, 6′ bond sites to produce 6-methyl-5-hepten-2-one (MHO), and also can produce pseudoionone due to the cleavage at 9, 10 (9′, 10′) bond sites. However, the cleavage at 5, 6 and/or 5′, 6′ bond sites may be more preferential than at 9, 10 (9′, 10′) bond sites because more abundant MHO was detected (Figure 6A). Other CCD1 enzymes from Arabidopsis, *Bixa Orellana, Zea mays*, and *Lycopersicon esculentum* can also cleave lycopene at 5, 6 and/or 5′, 6′ bond sites to form MHO (Vogel et al., 2008). However, MnCCD1 failed to produce MHO but produced pseudoionone due to the cleavage at 9, 10 (9′, 10′) sites (Qi et al., 2021). CCD1 from carrot didn’t use lycopene as a substrate (Yahyaa et al., 2013). OsCCD1 can cleave lycopene to produce pseudoionone and MHO, and geranial was also detected due to its cleavage at 7, 8 (7′, 8′) sites (Ilg et al., 2009). In some plants, there were two *CCD1* genes and they also showed different substrates and cleavage sites (Simkin et al., 2004a). These studies demonstrate the conserved cleavage activity of CCD1 enzymes as well as specificity in different plants.

In plants CCD1 was localized in the cytoplasm and can use various carotenoids and apocarotenoids as substrates (Auldridge et al., 2006b; Ilg et al., 2010), which can have great influences on the flavor, fragrance and quality. Loss of AtCCD1 results in increases in seed carotenoid contents but plants had no other changed phenotype (Auldridge et al., 2006b). In the maize endosperm, high levels of *CCD1* gene lead to dominant *White cap* mutant and deficient carotenoids in kernels (Timmermans et al., 2004). LeCCD1A and LeCCD1B in tomato can cleave lycopene that is responsible for the red fruit and produce C14 dialdehydes and pseudoionone and suppression of these two genes resulted in the decreased levels of beta-ionone and geranylacetone in fruits (Simkin et al., 2004a; Ilg et al., 2014). In our study, expression of *DoCCD1* gene in *N. benthamiana* leaves enhanced the level of beta-ionone (Figure 7), which can provide an alternative method for production of beta-ionone in plants.

To summarize, the present study confirmed the presence of beta-ionone in *D. officinale*, identified the CCD gene family members and analyzed their expression patterns. Further functional analyses demonstrate that DoCCD1 can catalyze different substrates to produce apocarotenoids. These data elucidate the potential role of DoCCD1 in the formation of beta-ionone in *D. officinale*.

## Data availability statement

The datasets presented in this study can be found in online repositories. The names of the repository/repositories and accession number(s) can be found in the article/Supplementary material.

## Author contributions

AL: conceptualization. YW and JX: experiments and data analysis. YW: writing—original draft. AL: writing—review and revising. All authors have read and agreed to the published version of the manuscript.

## Footnotes

1
https://ipsort.hgc.jp/index.html

2
https://phytozome.jgi.doe.gov/pz/portal.html

3
http://meme.nbcr.net/meme3/mme.html

4
http://www.ebi.ac.uk/interpro

5
http://gsds.cbi.pku.edu.cn/

6
https://rdrr.io/cran/pheatmap/

## Supplementary material

Supplementary Figure 1 The mass spectra of detected compounds.
