# Supplementary figures and images for: Identification of the carotenoid cleavage dioxygenase genes and functional analysis reveal DoCCD1 is potentially involved in beta-ionone formation in Dendrobium officinale
Source: Front Plant Sci. 2022 Aug 4;13:967819. doi: 10.3389/fpls.2022.967819 (PMC9387305; doi:10.3389/fpls.2022.967819)

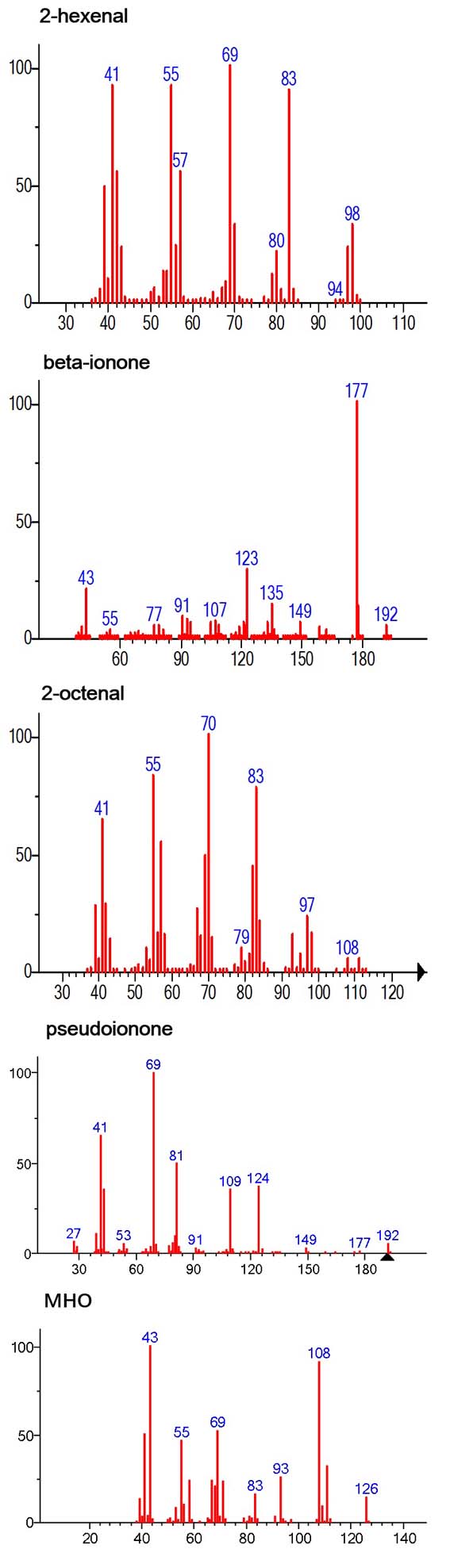

Supplement: Supplementary Figure 1 — The mass spectra of detected compounds. [file Image_1.JPEG]
